# Identification of crucial miRNAs and genes in esophageal squamous cell carcinoma by miRNA-mRNA integrated analysis

**DOI:** 10.1097/MD.0000000000016269

**Published:** 2019-07-05

**Authors:** Xiaowu Zhong, Guangcheng Huang, Qiang Ma, Hebin Liao, Chang Liu, Wenjie Pu, Lei Xu, Yan Cai, Xiaolan Guo

**Affiliations:** a Department of Clinical Laboratory, Affiliated Hospital of North Sichuan Medical College; b Translational Medicine Research Center; c Department of Laboratory Medicine, North Sichuan Medical College, Nanchong, Sichuan, China.

**Keywords:** bioinformatics, esophageal squamous cell carcinoma, miRNA, regulatory network

## Abstract

Esophageal squamous cell carcinoma (ESCC) is a malignancy that severely threatens human health and carries a high incidence rate and a low 5-year survival rate. MicroRNAs (miRNAs) are commonly accepted as a key regulatory function in human cancer, but the potential regulatory mechanisms of miRNA-mRNA related to ESCC remain poorly understood.

The GSE55857, GSE43732, and GSE6188 miRNA microarray datasets and the gene expression microarray datasets GSE70409, GSE29001, and GSE20347 were downloaded from Gene Expression Omnibus databases. The differentially expressed miRNAs (DEMs) and differentially expressed genes (DEGs) were obtained using GEO2R. Gene ontology (GO) and the Kyoto Encyclopedia of Genes and Genomes (KEGG) pathway enrichment analysis for DEGs were performed by Database for Annotation, Visualization and Integrated Discovery (DAVID). A protein–protein interaction (PPI) network and functional modules were established using the STRING database and were visualized by Cytoscape. Kaplan-Meier analysis was constructed based on The Cancer Genome Atlas (TCGA) database.

In total, 26 DEMs and 280 DEGs that consisted of 96 upregulated and 184 downregulated genes were screened out. A functional enrichment analysis showed that the DEGs were mainly enriched in the ECM-receptor interaction and cytochrome P450 metabolic pathways. In addition, *MMP9, PCNA, TOP2A, MMP1, AURKA, MCM2, IVL, CYP2E1, SPRR3, FOS, FLG, TGM1,* and *CYP2C9* were considered to be hub genes owing to high degrees in the PPI network. MiR-183-5p was with the highest connectivity target genes in hub genes. FOS was predicted to be a common target gene of the significant DEMs. Hsa-miR-9-3p, hsa-miR-34c-3p and FOS were related to patient prognosis and higher expression of the transcripts were associated with a poor OS in patients with ESCC.

Our study revealed the miRNA-mediated hub genes regulatory network as a model for predicting the molecular mechanism of ESCC. This may provide novel insights for unraveling the pathogenesis of ESCC.

## 1 Introduction

Esophageal carcinoma (EC) is the ninth most common cancer worldwide, with 572,034 new cases and 508,585 mortalities reported in 2018.^[1]^ The incidence and mortality of EC are rapidly growing worldwide in developing countries. Esophageal squamous cell carcinoma (ESCC) is the predominant histological type of EC, which is more prevalent in the developing world, with very high incidence areas found in East Asia, mainly in China.^[2,3]^ Epidemiological evidence suggests a different risk factor profile including cigarette smoking, alcohol consumption, highly salted meats, and so on.^[4,5]^ If earlier detection and aggressive medical and surgical treatment of EC could be achieved, then the five-year survival rates would exceed 80%.^[6,7]^ Unfortunately, because effective early diagnosis for EC remains elusive, EC often presents in an insidious and nonspecific manner, and the one-year survival rate remains lower than 15%.^[8–10]^ Therefore, the identification of a molecular mechanism and more efficient diagnostic methods for the early stages of EC can help to develop treatments for patients with ESCC.

During recent years, developments in molecular biology have provided gene expressions profiles for studying the pathogenesis of ESCC. Many scholars have carried out in-depth research on the pathogenesis of ESCC at the gene level, through which variety of significant genes were found. For instance, it was found that Programmed Cell Death 4 (PDCD4) is downregulated in ESCC.^[11]^ Phosphoinositide dependent kinase 1 (PDK1) is reported to be upregulated in ESCC, which is associated with a poor prognosis.^[12]^ In addition, microRNAs (miRNAs) are small noncoding RNA molecules of 20 to 25 nucleotides in length that participate in posttranscriptional regulation of gene expression. It has previously been demonstrated that miRNAs play an important role in tumor initiation and progression.^[13]^ In ESCC, miR-375 is frequently downregulated in cancer cells and functions as a tumor suppressor.^[14]^ MiR-183 might play an oncogenic role in suppressing apoptosis and promoting proliferation in ESCC by regulating the PDCD4 expression.^[15]^ It is known that miR-21 acts as an oncogenic miRNA in several types of cancer. The overexpression of miR-21 is related to advanced clinical stage, lymph node metastasis and a poor prognosis in ESCC.^[16,17]^ However, little is known regarding an integrated analysis of the miRNA-mRNA regulatory network in ESCC. Therefore, further study is imperative.

In this study, we analyzed differentially expressed miRNAs (DEMs) and genes (DEGs) between ESCC tumor tissues and normal tissue samples using bioinformatics methods. We performed functional and pathway enrichment analysis of DEGs, and established the miRNA-mRNA and protein–protein interactions (PPI) network to reveal regulatory mechanisms in ESCC. Here, we aimed to explore the main pathways and processes that are associated with ESCC and provide cancer biomarkers for diagnosis and therapy.

## 2 Materials and methods

### 2.1 Microarray data collection

The GSE55857, GSE43732, and GSE6188 miRNA expression profile data and three gene expression profiles (GSE70409, GSE29001, and GSE20347) were acquired from Gene Expression Omnibus (GEO: http://www.ncbi.nlm.nih.gov/geo/).^[18]^ For these datasets, only normal ESCC and ESCC tissue samples were selected for further analysis. Every included dataset contained more than 10 normal ESCC tissues and ESCC samples. Basic information on the miRNA and mRNA expression microarrays of ESCC is shown in Table 1.

**Table 1:**
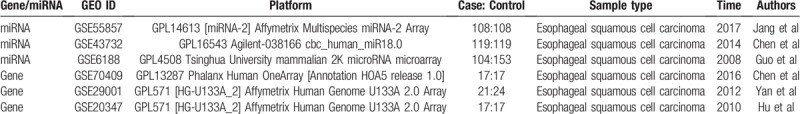
Basic information for the miRNA and mRNA expression microarrays for ESCC.

### 2.2 Identification of DEMs and DEGs

The differentially expressed miRNAs (DEMs) and differentially expressed genes (DEGs) were obtained from 2 groups of samples (ESCC vs normal ESCC tissues) in each GEO database. The raw data of miRNA and gene expression profiles were preprocessed by a way of GEO2R analysis (www.ncbi.nlm.nih.gov/geo/geo2r/). The adjusted *P* value < .01 and |logFC| > 2 were set as the cut-off criteria of the DEM and DEG analyses to identify a statistically significant difference.

**Figure 1:**
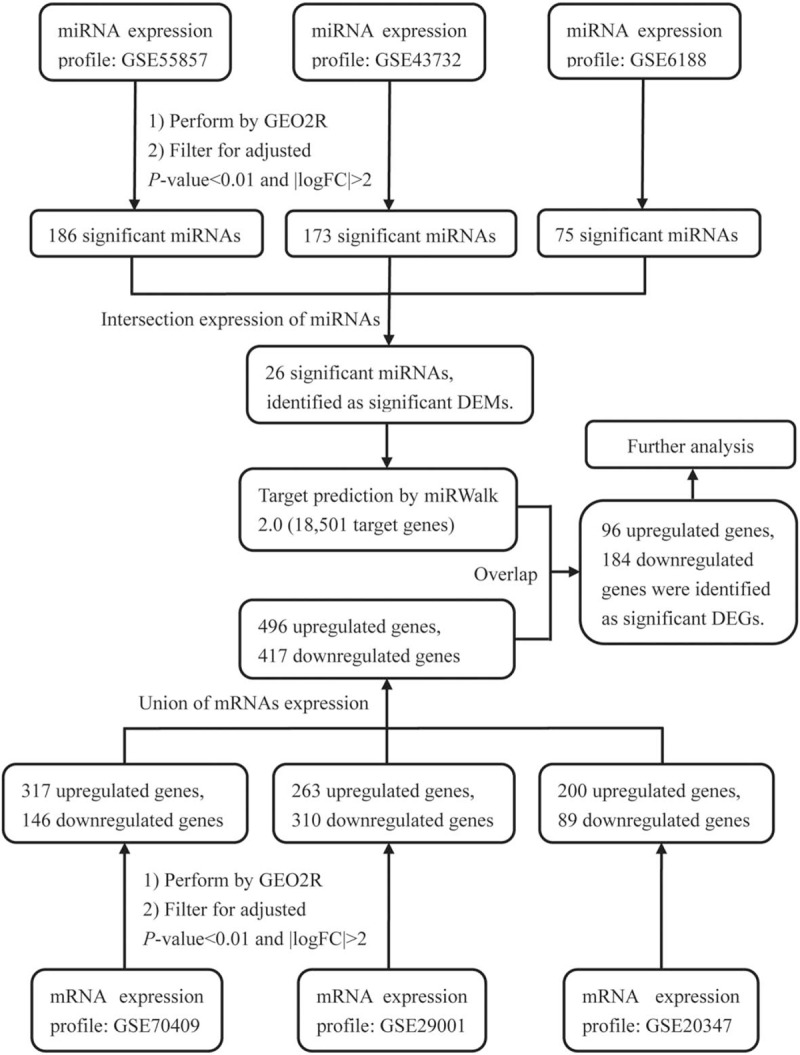
Flowchart of bioinformatics analysis.

The miRWalk 2.0 database (http://zmf.umm.uni-heidelberg.de/apps/zmf/mirwalk2/index.html) is a comprehensive atlas that supplies a large collection of predicted and experimentally verified miRNA-target interactions.^[19]^ We submitted the significant DEMs to miRwalk 2.0 and selected the data of their target mRNAs, which were based on experimental literature reports. Then, we extracted by intersecting the target mRNAs of the significant DEMs and the overlapping genes of the DEGs from the three GEO datasets. Finally, these genes were defined as significant DEGs (Fig. 1).

### 2.3 Gene ontology and KEGG pathway analysis

The Database for Annotation, Visualization and Integrated Discovery (DAVID, https://david-d.ncifcrf.gov/) is an online bioinformatics resource that provides functional and pathway enrichment analysis.^[20]^ Gene ontology (GO) and the Kyoto Encyclopedia of Genes and Genomes (KEGG) pathway enrichment analysis for DEGs was performed by with the DAVID online tool. A false discovery rate (FDR) <0.05 and gene count >2 were used as the cut-off criteria.

### 2.4 Construction of regulatory network

Search Tool for the Retrieval of Interacting Genes/Proteins (STRING, https://string-db.org/) is an online platform that is used to predict functional protein association networks.^[21]^ To construct protein–protein interactions (PPI) of DEGs, we submitted the DEGs to STRING, and only validated interactions that had a combined score >0.40, which was considered to be significant and the PPI was visualized.

Furthermore, the target genes of the significant DEMs were predicted by miRTarBase (http://mirtarbase.mbc.nctu.edu.tw/)^[22]^ and TargetScan (http://www.targetscan.org/).^[23]^ When the miRNAs shared a common target mRNA with the hub genes of DEGs, they might exist in a similar regulatory pathway. Finally, the miRNA-mRNA regulatory network depicted interactions between miRNAs and their potential targets in ESCC were visualized by using Cytoscape 3.7.0.^[24]^

### 2.5 Survival analysis

A Kaplan–Meier analysis was performed using OncoLnc (http://www.oncolnc.org/),^[25]^ in which boxplots were employed to visualize the expression level of all prognosis-related genes between ESCC and normal tissues, that was based on data from the TCGA database (https://cancergenome.nih.gov/). Data from 144 ESCC patients were found in the TCGA database. Among them, 117 patients were male, and 27 were female. Among the 144 available clinical outcome events, 85 patients were living and 59 were deceased when the follow-up period ended. The *P* value < .05 was considered to be statistically significant.

## 3 Results

### 3.1 Identification of DEMs and DEGs

**Figure 2:**
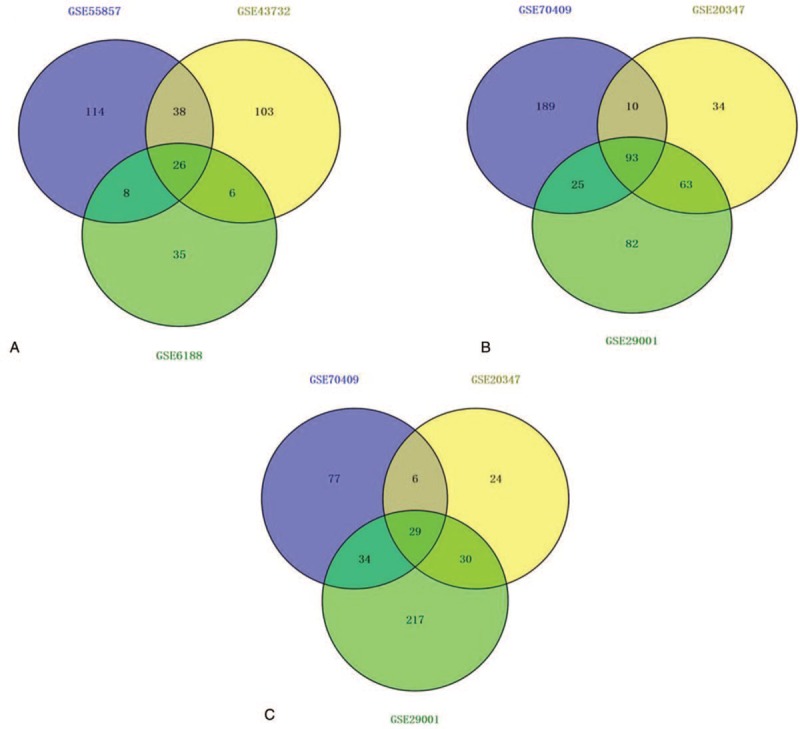
Identification of differentially expressed miRNAs (DEMs) and genes (DEGs). (A) Identification of DEMs; (B) Identification of upregulated DEGs; (C) Identification of downregulated DEGs.

A total of 186, 173, and 75 DEMs were identified from the GSE55857, GSE43732, and GSE6188 datasets, respectively. As shown in Figure 2A, 26 DEMs were screened out in all 3 datasets, so that they could be identified as significant DEMs, which were hsa-miR-18b-5p, hsa-miR-503-5p, hsa-miR-9-3p, hsa-miR-133a-3p, hsa-miR-34b-5p, hsa-miR-34c-5p, hsa-miR-339-3p, hsa-miR-375, hsa-miR-133b, hsa-miR-203a, hsa-miR-150-5p, hsa-miR-493-3p, hsa-miR-18a-3p, hsa-miR-130b-3p, hsa-miR-431-5p, hsa-miR-7-5p, hsa-miR-382-5p, hsa-miR-182-3p, hsa-miR-424-5p, hsa-miR-146b-5p, hsa-miR-196a-3p, hsa-miR-183-5p, hsa-miR-1-3p, hsa-miR-196b-3p, and hsa-miR-497-5p.

Compared with the normal samples, a total of 496 upregulated DEGs and 417 downregulated DEGs were identified in the ESCC samples (Fig. 2B and C). We obtained 18,501 target genes of the 26 significant DEMs from the miRWalk 2.0 database. The intersection number of these target genes and the upregulated DEGs that appeared in the three datasets was 96, while the intersection number of these candidate genes and the downregulated DEGs which appeared in three datasets was 184. Therefore, the 96 upregulated and 184 downregulated genes were identified as the final sets of significant DEGs.

### 3.2 Functional annotation analysis

GO ontology contains three terms: molecular function (MF), cellular component (CC) and biological process (BP). In MF ontology, the most significant GO terms for upregulated significant DEGs were the extracellular matrix structural constituent, platelet-derived growth factor binding and metalloendopeptidase activity. Whereas the terms for the downregulated significant DEGs, the terms were iron ion binding and monooxygenase activity. In CC ontology, the extracellular region, the proteinaceous extracellular matrix, the extracellular matrix, the collagen trimer, the extracellular space and the endoplasmic reticulum lumen were significantly enriched GO terms for upregulated genes, while the extracellular exosome, the cornified envelope and the extracellular space were significantly enriched GO terms for downregulated genes. In BP ontology, the upregulated genes were mainly enriched in collagen catabolic process, extracellular matrix organization, skeletal system development, extracellular matrix disassembly and collagen fibril organization. The downregulated genes were mainly enriched in keratinocyte differentiation, keratinization, peptide cross-linking, epithelial cell differentiation and epidermis development.

Moreover, 4 KEGG pathways were overrepresented in the upregulated genes, including ECM-receptor interaction, amoebiasis, focal adhesion and protein digestion and absorption, and only 1 pathway (drug metabolism-cytochrome P450) was identified in the downregulated genes. The detailed results are presented in Table 2.

**Table 2:**
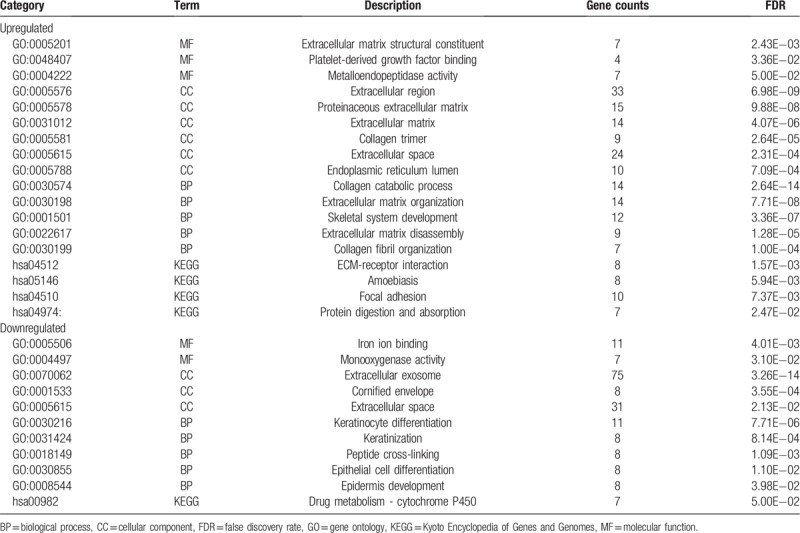
Significantly enriched GO biological process terms and KEGG pathways.

### 3.3 PPI network

**Figure 3:**
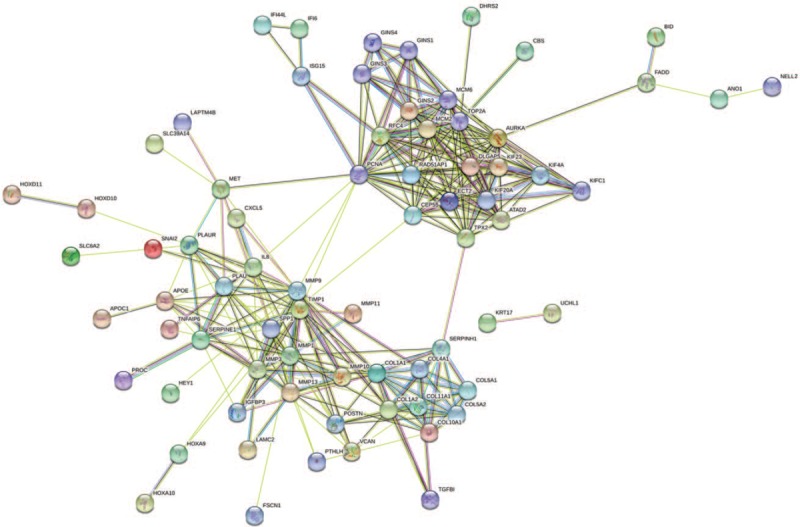
PPI networks of significantly upregulated DEGs. Colored nodes: query proteins and first shell of interactors, white nodes: second shell of interactors; Blue-green line: known interactions from curated databases, purple line: known interactions from experimentally determined, green line: predicted interactions form gene neighborhood, red line: predicted interactions form gene fusions, dark blue: predicted interactions form gene co-occurrence, yellow line: interactions form textmining, black line: interactions form co-expression, light blue: interactions form protein homology.

**Figure 4:**
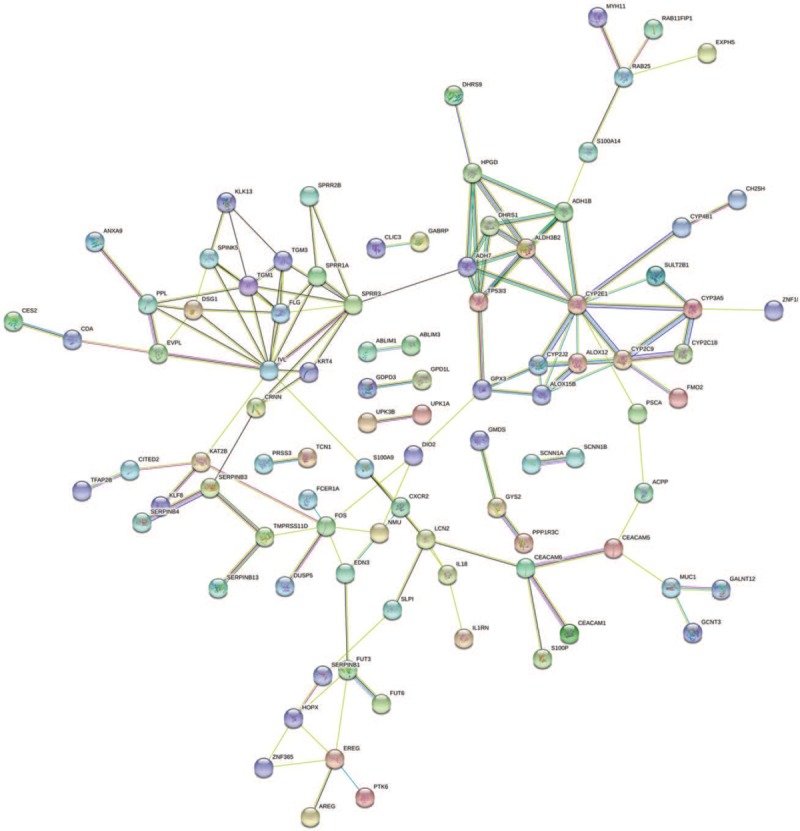
PPI networks of significantly downregulated DEGs. Colored nodes: query proteins and first shell of interactors, white nodes: second shell of interactors; Blue-green line: known interactions from curated databases, purple line: known interactions from experimentally determined, green line: predicted interactions form gene neighborhood, red line: predicted interactions form gene fusions, dark blue: predicted interactions form gene co-occurrence, yellow line: interactions form textmining, black line: interactions form co-expression, light blue: interactions form protein homology.

The PPI network of DEGs was established on the basis of STRING. The 103 nodes and 316 edges in total constituted the PPI network of significantly upregulated DEGs (Fig. 3). The network that significantly downregulated DEGs was composed of 188 nodes and 138 edges (Fig. 4).

The edge information plays an important role in the identification of hub genes in a PPI network. We used the parameter“degree” to calculate the edge counts of every single gene in a PPI network. The top 5% degree genes are shown in Table 3, which were assessed as hub genes. Thirteen genes in the PPI network were selected as hub genes in ESCC. These hub genes might play crucial roles in ESCC.

**Table 3:**
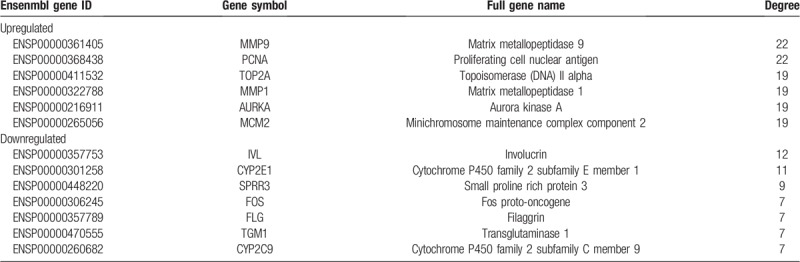
Top 5% hub genes in the PPI networks.

### 3.4 miRNA–mRNA interaction network

**Figure 5:**
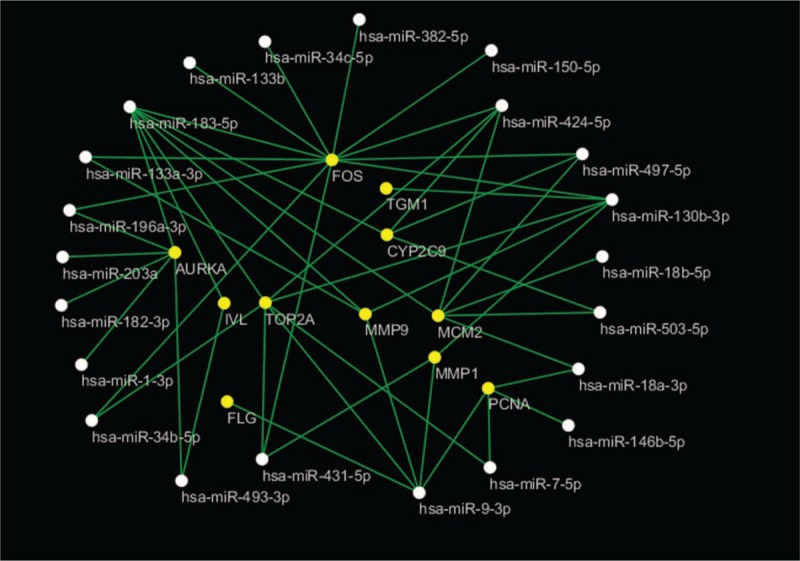
The miRNA-mRNA regulatory network. White nodes, miRNA; Green nodes, mRNA.

To further understand the regulatory relationship between 26 significant DEMs and hub genes, the miRNA-mRNA regulation network was established and is shown in Figure 5. There were no hub genes that could be the target genes of hsa-miR-339-3p, hsa-miR-375 or hsa-miR-196b-3p. MiR-183-5p was with the highest connectivity target genes, targeting 7 hub genes (upregulated: MMP9, TOP2A, AURKA, and MCM2; downregulated: IVL, FOS, and CYP2C9). Five hub genes could be target genes of hsa-miR-9-3p and hsa-miR-130b-3p. Moreover, several genes were predicted to be common targets of different miRNAs. For instance, FOS was predicted as a common target of hsa-miR-133a-3p, hsa-miR-34b-5p, hsa-miR-34c-5p, hsa-miR-133b, hsa-miR-150-5p, hsa-miR-130b-3p, hsa-miR-431-5p, hsa-miR-382-5p, hsa-miR-424-5p, hsa-miR-196a-3p, hsa-miR-183-5p, and hsa-miR-497-5p.

### 3.5 Stage and survival analysis of miRNA/mRNA in ESCC

We investigated the miRNAs expression level of 26 significant DEMs in the TCGA dataset, and found that 23 miRNAs were consistent with the results from GEO databases. However, the expression levels of hsa-miR-203a, hsa-miR-182-3p, and hsa-miR-1-3p were not significantly different between the ESCC samples and normal ESCC tissues. Using the TCGA ESCA dataset, we assessed the relationship of miRNAs with the clinical TNM stages. Detailed results of the significant DEMs are presented in Table 4.

**Table 4:**
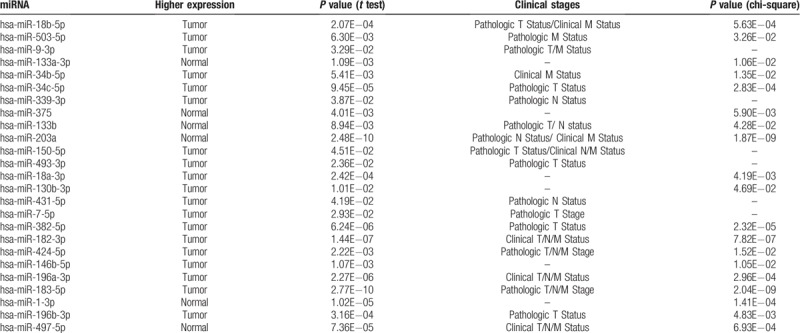
Results of the significant DEMs expression validation and the relationship with ESCC clinical stages in the TCGA dataset.

**Figure 6:**
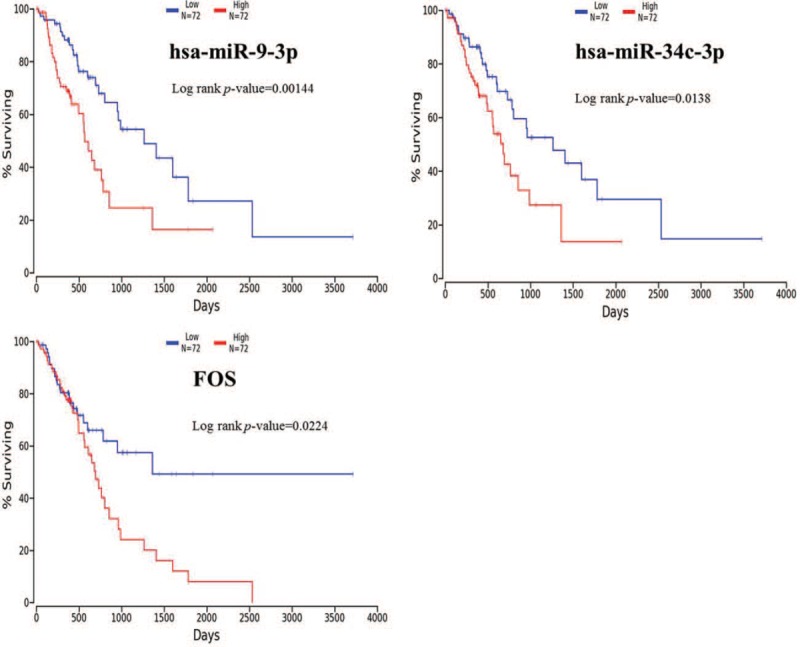
Prognostic values of hsa-miR-9-3p, hsa-miR-34c-3p and FOS for overall survival in ESCC patients. ESCC patients were divided into low- and high-expression groups. N represents the number of patients in each group.

Based on the TCGA survival data, survival analysis using the Kaplan–Meier method and the log-rank test was conducted. The results indicated that hsa-miR-9-3p, hsa-miR-34c-3p and FOS were related to overall survival (OS) in ESCC patients (Fig. 6). Higher expression of the transcript was associated with the poor OS in patients with ESCC.

## 4 Discussion

ESCC is one of the most lethal cancers and is a public health issue of great concern worldwide.^[26]^ Despite many advances in diagnosis and treatment, there is still no effective treatment for ESCC, and survival remains very poor.^[27]^ Thus, the molecular mechanisms that are involved in the progress of ESCC have not been clarified. Therefore, it is crucial to study the mechanism and identify the molecular targets for diagnosis and treatment. In the present study, we identified 26 significant DEMs, 96 upregulated DEGs, and 184 downregulated DEGs. The results of the functional enrichment analysis indicated that significant DEGs were related to the ECM-receptor interaction and cytochrome P450 pathways in ESCC. Thirteen genes in the PPI network were selected as hub genes in ESCC. Importantly, hsa-miR-9-3p, hsa-miR-34c-3p and FOS were validated and were found to be correlated with tumor stages and survival, which meant they could not only regulate cellular process but could also be of valuable in clinical practice.

As was suggested by the functional enrichment analysis of significant DEGs, we found that the DEGs were mainly enriched in biological processes including, the ECM-receptor interaction and the cytochrome P450 metabolic pathways, and so on. ECM-receptor interaction was a chief contributor of cancer progression.^[28]^ Cytochrome P450 levels showed significant differences between ESCC patients and healthy subjects and may contribute to the development of ESCC.^[29]^ The genetic polymorphism RsaI/PstI in cytochrome P450 2E1 (CYP2E1) was a risk factor for EC. It demonstrated that CYP2E1 was a genetic determinant in the development of ESCC.^[30]^ CYP2C9 expression was relevant for high Ki-67 labeling indices in EC. Moreover, inhibition of cytochrome P450 2C9 (CYP2C9) could affect the tumor cell proliferation in early EC development.^[31]^

A growing body of evidence suggests that miRNAs have significant roles in human tumorigenesis, tumor progression and metastasis.^[32]^ MiRNAs are unique in their ability to regulate mRNA, which is more than 60% of protein-coding genes.^[33]^ The aberrant expression of miRNAs in ESCC causes destruction to the miRNA-regulated mRNA networks and can function as tumor suppressors or oncogenes.^[34]^ Therefore, the identification of the miRNA-mRNA regulatory network is important for further research concerning ESCC. Compared with normal samples, a microarray analysis identified 26 significant DEMs in our results. MiR-18a/b,^[35,36]^ miR-503,^[37]^ miR-9,^[38]^ miR-133a/b,^[39,40]^ miR-34b/c,^[41,42]^ miR-375,^[43]^ miR-203a,^[44]^ miR-150,^[45]^ miR-130b,^[46]^ miR-7,^[47]^ miR-382,^[48]^ miR-424,^[49]^ miR-146b,^[50]^ miR-196a,^[51]^ miR-183,^[15,52]^, and miR-1^[53]^ were reported in ESCC studies, and mostly involved in ESCC pathogenesis. MiR-18a overexpression was positively correlated with the stage that promoted the expression Cyclin D1 regulating PTEN-PI3K-AKT-mTOR signaling axis in ESCC cells. MiR-150 plays an oncogenic role in ESCC. MiR-150 promots ESCC cell migration and invasion by directly targeting ZEB1, SPOCK1 and Gli1.^[45,54,55]^ MiR-133a, miR-133b and miR-375 are known diagnostic and prognostic markers that are associated with tumor suppressor miRNAs. MiR-133a, miR-133b and miR-375 are frequently downregulated in ESCC and are closely related to advanced clinical stage, tumor metastasis and poor prognosis. MiR-133a can suppress the migration and invasion of ESCC cells by targeting Sox4 and the EMT process. MiR-133b can regulate metastases of ESCC by affecting the MAPK/ERK and PI3K/AKT signaling pathways by targeting EGFR. MiR-375 is involved in the development and progression of ESCC by repressing metadherin expression.^[39,40,43]^ In this study, higher expression levels of hsa-miR-9-3p and hsa-miR-34c-3p were found to be associated with poor OS in patients with ESCC. It was reported that miR-9 induces epithelial-mesenchymal transition (EMT) in ESCC, which is a key event in tumor metastasis. High plasma miR-9 concentrations are significantly correlated with poor tumor differentiation.^[38,56]^ MiR-34b and miR-34c are located on intron 1 and exon 2 of the common primary transcript. MiR-34b and miR-34c expression levels were significantly higher in ESCC in the corresponding normal samples. Inhibiting the expression of miR-34b or miR-34c in ESCC cells may suppress cell growth in vitro. However, it may inactivate the p53-miR-34 pathway. Taken together, these significant DEMs provide potential biomarkers and molecular mechanisms for the diagnosis and therapy of ESCC.

Global alterations of miRNA and mRNA expression are involved in the regulatory mechanisms of the development and progression of ESCC.^[57]^ Through PPI analyses, we found that the 13 mRNAs that were assessed as hub genes might play crucial roles in ESCC. Matrix metalloproteinases (MMPs) are strongly expressed in the cytoplasm of cancer cells, especially in the invasive margin, and are weakly expressed in stromal cells. MMP9 expression is positively associated with poor tumor cell differentiation.^[58]^ MMP1 promotes tumor growth and metastasis when the PI3K/AKT pathway is inhibited by LY294002.^[59]^ Proliferating Cell Nuclear Antigen (PCNA) expression can serve as an independent prognostic factor of EC.^[60]^ The c-FOS which belongs to FOS protein family that is involved in the transcriptional regulation of ID1, responds to etoposide in ESCC cells.^[61]^ Topoisomerase IIα (TOP2A) is associated with active cell proliferation of mammalian cells, and have reported expression levels and prognostic value in ESCC patients.^[62]^ AURKA can directly interacts with β-catenin enhances tumor cell invasion and metastatic in vitro and in vivo, and upregulates MMP-2 expression through activating AKT/NF-κB pathway in ESCC cells.^[63,64]^ Minichromosome maintenance protein 2 (MCM2) expression is useful marker than Ki-67 in predictingtumor aggressiveness and prognostic value in ESCC.^[65]^ Involucrin (IVL) IVL and filaggrin (FLG) are major components of the cornified envelope and are considered to be appropriate markers for terminal differentiation.^[66,67]^ Small proline-rich repeat protein 3 (SPRR3) was downregulated and involved in radiosensitivity in ESCC patients.^[68]^ Transglutaminase-1 (TGM1) can suppress the Wnt signaling pathway and involve in development of gastric cancer.^[69]^ By constructing a miRNA-mRNA network, miR-183 was shown to have the highest connectivity to target genes and to target, MMP9, TOP2A, AURKA, MCM2, IVL, FOS, and CYP2C9. MiR-183 might play an oncogenic role by suppressing apoptosis and promoting proliferation in ESCC by regulating PDCD4 expression.^[15,52]^ Five hub genes could be the target genes of hsa-miR-9-3p and hsa-miR-130b-3p. Moreover, the FOS gene was predicted to be a common target of different miRNAs. FOS and miR-34c had higher expression levels, which were found to be associated with poor OS in patients with ESCC. Interestingly, FOS was a target gene of hsa-miR-34c-3p determined by both miRTarBase and TargetScan. It revealed that miR-34c might be involved in the regulation of FOS. Further experimental studies are required to test our results. We identified that the hub genes that miRNAs may interact with might play important roles in ESCC. In addition, the identification of the miRNA-mRNA regulation network provided new insights into ESCC oncogenesis.

## 5 Conclusion

In this study, 26 DEMs and 280 DEGs that consisted of 96 upregulated and 184 downregulated genes were identified. Hsa-miR-9-3p, hsa-miR-34c-3p and FOS were found to be related to OS in ESCC patients. The higher expression levels of the transcript were associated with poor OS in patients with ESCC. The present study provides potential biomarkers and molecular mechanisms for the diagnosis and therapy of ESCC and provides valuable clues for further research.

## Author contributions

**Data curation:** Guangcheng Huang.

**Formal analysis:** Guangcheng Huang, Wenjie Pu.

**Funding acquisition:** Xiaowu Zhong.

**Methodology:** Hebin Liao, Chang Liu.

**Project administration:** Xiaowu Zhong, Xiaolan Guo.

**Resources:** Lei Xu.

**Software:** Qiang Ma, Chang Liu, Yan Cai.

**Supervision:** Yan Cai, Xiaolan Guo.

**Writing – original draft:** Xiaowu Zhong.

**Writing – review & editing:** Xiaolan Guo.
